# Supplementary material for: A Likelihood Approach for Real-Time Calibration of Stochastic Compartmental Epidemic Models
Source: PLoS Comput Biol. 2017 Jan 17;13(1):e1005257. doi: 10.1371/journal.pcbi.1005257 (PMC5240920; doi:10.1371/journal.pcbi.1005257)
Supplement: S1 File — (TAR.GZ) [file pcbi.1005257.s014.tar.gz › HSPH_Online-SI-Revision/IPoi09/n1-extreme/n1-extreme_BENCH_table0.pdf]

1	R0	{2.95019, 14.2854}	{2.86728, 14.694}	{{2.86193, 14.6465}, {2.86193, 14.6465}}	{2.89129, 2.86012}	inf	302.	245.672	{190., 298.}	19.3139	0.105837	newly	137.	111.422	{84., 139.}	19.6738	0.104424	Reff	0.882107	0.95799	{0.515147, 1.62558}	42.6945	ar	924.	930.234	{916., 944.}	1.15835
2	R0	{2.67565, 16.946}	{2.91024, 18.9193}	{{2.8568, 18.4249}, {2.97157, 19.9817}}	{8.76769, 11.6445}	inf	233.	217.931	{164., 279.}	16.7442	0.080093	newly	80.	74.3472	{50., 99.}	20.3646	0.0984044	Reff	0.559211	0.558453	{0.308061, 0.864295}	32.9025	ar	926.	931.125	{915., 944.}	1.03942
3	R0	{2.40066, 16.6543}	{2.87176, 19.0146}	{{2.75274, 17.3228}, {2.93978, 19.9817}}	{19.6239, 14.1724}	inf	237.	255.111	{199., 318.}	17.9208	0.082636	newly	91.	92.5972	{69., 112.}	14.6062	0.0684913	Reff	1.01788	1.05274	{0.604517, 1.66712}	35.6165	ar	916.	931.083	{911., 951.}	2.01055
4	R0	{1.9294, 15.4361}	{2.75459, 19.5797}	{{2.59755, 18.2464}, {2.81635, 19.984}}	{42.7694, 26.8434}	inf	135.	244.117	{188., 293.}	81.52	0.253485	newly	58.	82.8701	{62., 100.}	44.3574	0.154703	Reff	1.07853	1.0248	{0.557614, 1.54175}	30.6952	ar	796.	919.182	{896., 938.}	15.4751
5	R0	{2.92859, 19.5393}	{1.30222, 1.35944}	{{1.30222, 1.35944}, {1.30222, 1.35944}}	{55.5342, 93.0425}	inf	261.	0.	{0., 0.}	100.	3.41664	newly	98.	0.	{0., 0.}	100.	2.99123	Reff	1.27329	0.604143	{0.5, 0.5}	200.	ar	927.	1.	{1., 1.}	99.8921
6	R0	{1.79622, 12.3148}	{2.63829, 19.3496}	{{2.46976, 17.5057}, {2.78218, 19.9254}}	{46.8802, 57.1244}	inf	97.	205.896	{153., 258.}	114.295	0.323995	newly	44.	70.8806	{52., 87.}	62.8562	0.20506	Reff	0.941219	0.781158	{0.439271, 1.21612}	38.7164	ar	734.	904.985	{875., 931.}	23.295
7	R0	{2.45855, 17.1776}	{2.81224, 14.399}	{{2.52588, 12.3139}, {2.96947, 15.6207}}	{14.5145, 16.1758}	inf	208.	182.556	{118., 240.}	22.8098	0.116661	newly	81.	78.9722	{49., 111.}	26.3032	0.119408	Reff	0.98342	0.521826	{0.287915, 0.793581}	51.5764	ar	871.	922.944	{899., 942.}	5.98291
8	R0	{2.20009, 15.519}	{2.51802, 19.2729}	{{2.42205, 19.1373}, {2.64373, 19.7855}}	{14.4507, 24.1889}	inf	171.	133.323	{79., 192.}	28.9429	0.161313	newly	72.	44.8	{25., 69.}	38.5897	0.241212	Reff	0.708429	0.50552	{0.308025, 0.723734}	39.5328	ar	839.	893.569	{871., 910.}	6.50408
9	R0	{2.06493, 9.6414}	{2.89436, 15.0485}	{{2.84915, 14.6465}, {2.96947, 15.6207}}	{40.1677, 56.0817}	inf	125.	229.5	{169., 283.}	83.6	0.255734	newly	96.	98.5417	{68., 132.}	20.5584	0.0894889	Reff	1.08409	0.652991	{0.364672, 0.973056}	48.1172	ar	777.	934.25	{919., 948.}	20.2381
10	R0	{2.60775, 19.6936}	{2.42149, 19.4437}	{{2.42205, 19.4475}, {2.42205, 19.4475}}	{7.14251, 1.26903}	inf	215.	156.933	{94., 211.}	29.0078	0.174931	newly	62.	55.05	{30., 83.}	28.0914	0.150939	Reff	0.904889	0.762305	{0.399638, 1.25704}	42.7637	ar	889.	878.933	{845., 903.}	1.9835
11	R0	{1.99168, 8.13555}	{2.88527, 14.8402}	{{2.81617, 13.9425}, {2.96481, 15.2798}}	{44.866, 82.4124}	inf	102.	179.566	{116., 244.}	76.0449	0.23022	newly	81.	73.7237	{46., 109.}	25.7472	0.126142	Reff	0.938081	0.491691	{0.293516, 0.684001}	54.4149	ar	772.	930.658	{916., 947.}	20.5515
12	R0	{1.79304, 9.18941}	{2.80021, 15.7231}	{{2.7499, 15.0558}, {2.8939, 16.8013}}	{56.1711, 71.0998}	inf	134.	240.891	{179., 301.}	81.4249	0.250967	newly	109.	107.625	{84., 130.}	14.0482	0.064086	Reff	1.0059	1.21314	{0.580793, 1.98339}	45.0578	ar	725.	926.484	{905., 945.}	27.7909
13	R0	{1.84016, 18.3431}	{2.26501, 19.3332}	{{2.25962, 19.0355}, {2.30371, 19.6625}}	{23.0875, 5.52632}	inf	100.	161.641	{107., 209.}	63.8281	0.20532	newly	41.	55.3125	{35., 75.}	43.2927	0.155819	Reff	1.13354	0.805332	{0.50912, 1.19464}	35.1179	ar	762.	854.922	{825., 890.}	12.1945
14	R0	{1.94221, 9.85971}	{2.98398, 18.7214}	{{2.96797, 18.4042}, {2.99088, 18.9406}}	{53.6385, 89.8777}	inf	139.	211.467	{137., 288.}	53.7458	0.174116	newly	92.	72.6933	{45., 100.}	26.058	0.142152	Reff	0.594316	0.601016	{0.299765, 1.12555}	44.7035	ar	805.	935.24	{917., 950.}	16.1789
15	R0	{1.78578, 12.0541}	{2.28151, 19.2031}	{{2.26161, 19.0355}, {2.29738, 19.3849}}	{27.7601, 59.3078}	inf	118.	153.103	{94., 201.}	39.6436	0.168553	newly	65.	53.5294	{33., 76.}	24.4796	0.154158	Reff	0.878604	0.803234	{0.464071, 1.31626}	32.3986	ar	732.	843.515	{824., 884.}	18.0669
16	R0	{1.94463, 15.6503}	{2.7987, 15.6412}	{{2.66419, 14.2056}, {2.8939, 16.8013}}	{43.9196, 3.91384}	inf	143.	183.292	{120., 260.}	36.6841	0.130304	newly	51.	72.7222	{48., 108.}	47.8214	0.157555	Reff	0.958703	0.51835	{0.316239, 0.791971}	50.5902	ar	757.	925.625	{909., 943.}	22.2754
17	R0	{2.08901, 16.4314}	{2.63595, 19.5837}	{{2.46976, 17.5057}, {2.66225, 19.9254}}	{26.182, 19.1849}	inf	160.	203.778	{132., 256.}	38.316	0.162954	newly	61.	73.3056	{52., 93.}	31.3297	0.156242	Reff	1.04868	1.12827	{0.563114, 2.01188}	42.3752	ar	826.	905.667	{881., 927.}	9.64487
18	R0	{2.76419, 18.56}	{2.82638, 16.2934}	{{2.7499, 15.3124}, {2.96797, 18.1089}}	{2.49783, 12.3352}	inf	275.	195.246	{130., 265.}	29.8294	0.169575	newly	103.	75.7231	{48., 110.}	29.9477	0.170464	Reff	0.931532	0.536537	{0.320541, 0.860618}	49.724	ar	920.	928.492	{914., 944.}	1.28094
19	R0	{2.3821, 17.1399}	{2.78485, 14.3756}	{{2.52368, 12.2207}, {2.96947, 15.6207}}	{17.0413, 16.128}	inf	229.	185.333	{122., 259.}	25.0988	0.13371	newly	77.	79.1905	{49., 115.}	28.86	0.125125	Reff	1.07433	0.565287	{0.295376, 1.01779}	54.5507	ar	883.	922.667	{896., 946.}	4.71157
20	R0	{2.78314, 19.6127}	{2.64715, 19.8154}	{{2.64373, 19.7855}, {2.66225, 19.9254}}	{4.88619, 1.08195}	inf	236.	173.806	{108., 251.}	29.0019	0.169321	newly	84.	56.3611	{32., 86.}	34.3585	0.208373	Reff	0.411905	0.67123	{0.348972, 1.23991}	70.6278	ar	943.	908.375	{894., 928.}	3.67179
21	R0	{1.85124, 14.6794}	{2.422, 18.8706}	{{2.24732, 17.1868}, {2.50427, 19.5287}}	{30.8314, 28.5515}	inf	97.	201.953	{144., 252.}	108.747	0.310814	newly	43.	73.6719	{55., 90.}	72.202	0.229083	Reff	1.20516	1.03308	{0.605943, 1.70356}	30.1325	ar	730.	882.297	{857., 908.}	20.8626
22	R0	{2.1028, 14.9852}	{2.96984, 18.4894}	{{2.96797, 18.1089}, {2.99088, 18.9406}}	{41.2329, 23.3842}	inf	167.	168.041	{107., 248.}	26.4849	0.115397	newly	63.	57.4595	{35., 90.}	28.7859	0.136704	Reff	0.738083	0.398299	{0.247444, 0.592194}	48.8723	ar	840.	934.851	{921., 948.}	11.2918
23	R0	{1.86601, 10.2157}	{2.89531, 17.2669}	{{2.78517, 15.7559}, {2.98558, 18.4749}}	{55.1605, 69.0231}	inf	118.	244.042	{193., 290.}	106.815	0.309809	newly	79.	91.9014	{66., 113.}	22.9988	0.090257	Reff	1.02071	0.686859	{0.399511, 0.973024}	40.8055	ar	747.	931.845	{916., 944.}	24.745
24	R0	{2.34032, 10.0992}	{2.86193, 14.6465}	{{2.86193, 14.6465}, {2.86193, 14.6465}}	{22.288, 45.0263}	inf	202.	245.033	{191., 298.}	28.1691	0.113758	newly	137.	112.016	{84., 139.}	19.2892	0.102616	Reff	0.840175	0.971836	{0.546629, 1.50538}	43.9243	ar	887.	929.213	{915., 944.}	4.77018
25	R0	{1.91548, 15.3401}	{2.63276, 19.149}	{{2.46976, 17.5057}, {2.79269, 19.984}}	{37.4462, 24.8294}	inf	146.	212.789	{165., 268.}	52.46	0.189081	newly	68.	74.1972	{54., 97.}	21.6239	0.0964092	Reff	1.10523	0.91103	{0.51865, 1.5897}	40.6852	ar	765.	907.761	{887., 929.}	18.6615
26	R0	{2.75283, 10.6721}	{2.78803, 15.7647}	{{2.78517, 15.7559}, {2.78517, 15.7559}}	{1.285, 47.7189}	inf	255.	234.032	{177., 286.}	15.2194	0.0944286	newly	153.	98.5873	{64., 122.}	35.5639	0.213975	Reff	0.743264	0.974107	{0.509686, 1.38423}	49.0952	ar	926.	923.778	{911., 938.}	1.14848
27	R0	{2.08367, 19.7849}	{2.55755, 19.355}	{{2.46976, 18.9032}, {2.64373, 19.7855}}	{22.7424, 2.21795}	inf	158.	171.366	{109., 233.}	26.199	0.109346	newly	54.	57.8028	{34., 83.}	28.8993	0.122523	Reff	0.927233	0.673845	{0.398169, 1.13353}	42.2141	ar	833.	895.803	{871., 921.}	7.53935
28	R0	{2.64334, 17.1765}	{2.7995, 15.5804}	{{2.7499, 15.0981}, {2.8939, 16.2348}}	{5.90773, 9.43514}	inf	230.	204.478	{144., 262.}	17.9017	0.09085	newly	99.	83.7536	{54., 116.}	21.9587	0.11521	Reff	0.734849	0.647116	{0.373986, 1.06307}	39.852	ar	934.	923.174	{911., 938.}	1.43841
29	R0	{2.87008, 13.3872}	{2.64484, 19.7939}	{{2.64373, 19.7855}, {2.64373, 19.7855}}	{7.84782, 47.8569}	inf	267.	206.869	{163., 250.}	22.5579	0.126396	newly	113.	71.2623	{53., 89.}	36.936	0.214711	Reff	0.608457	0.9036	{0.444147, 1.48049}	58.6843	ar	941.	908.475	{886., 928.}	3.47381
30	R0	{2.06105, 19.6275}	{2.42071, 19.4476}	{{2.42205, 19.4475}, {2.42205, 19.4475}}	{17.4504, 0.916586}	inf	100.	108.22	{46., 181.}	41.4746	0.17716	newly	41.	35.4576	{13., 66.}	43.2823	0.238383	Reff	0.715184	0.527748	{0.318199, 0.750836}	41.6739	ar	747.	878.847	{845., 905.}	17.6503
31	R0	{2.30764, 13.435}	{2.86691, 14.8681}	{{2.84915, 14.6465}, {2.90379, 15.0981}}	{24.2355, 10.6666}	inf	231.	158.029	{83., 241.}	35.8442	0.212674	newly	94.	65.	{35., 107.}	38.541	0.228189	Reff	0.717676	0.452663	{0.263298, 0.63821}	52.1399	ar	877.	932.129	{918., 944.}	6.28604
32	R0	{2.30565, 11.9237}	{2.81856, 15.4763}	{{2.7499, 15.0558}, {2.95376, 15.963}}	{22.2456, 29.7942}	inf	219.	231.582	{164., 292.}	18.8032	0.0812627	newly	128.	100.239	{70., 124.}	23.4841	0.124085	Reff	0.788532	0.859525	{0.390334, 1.61836}	46.0747	ar	853.	927.761	{912., 943.}	8.7645
33	R0	{2.24031, 18.582}	{2.42192, 19.4044}	{{2.41419, 19.391}, {2.42625, 19.4475}}	{8.10624, 4.42578}	inf	205.	180.235	{129., 224.}	17.5179	0.0919976	newly	78.	63.1176	{44., 84.}	22.6621	0.121645	Reff	1.10895	0.94852	{0.520741, 1.77052}	36.9507	ar	864.	878.456	{852., 903.}	2.26545
34	R0	{2.33306, 15.9759}	{2.95614, 18.2046}	{{2.83341, 16.5525}, {2.99088, 18.9406}}	{26.7067, 13.9505}	inf	204.	257.693	{217., 305.}	28.7124	0.108146	newly	96.	95.52	{74., 117.}	13.2222	0.0588395	Reff	0.874898	0.853348	{0.414996, 1.36982}	38.0119	ar	854.	936.253	{927., 947.}	9.63154
35	R0	{2.48357, 18.3573}	{2.79347, 15.5259}	{{2.66419, 14.2056}, {2.8939, 16.8013}}	{12.4782, 15.4236}	inf	218.	215.071	{158., 272.}	17.7785	0.0822771	newly	79.	88.5429	{59., 115.}	24.34	0.099122	Reff	1.0282	0.629468	{0.369092, 0.931836}	44.409	ar	835.	924.	{906., 941.}	10.6587
36	R0	{2.59548, 19.9046}	{2.79248, 19.7513}	{{2.7811, 19.6712}, {2.81635, 19.898}}	{7.8806, 0.802166}	inf	240.	229.632	{166., 289.}	17.7267	0.0964772	newly	83.	79.5735	{50., 100.}	19.3303	0.100507	Reff	0.931777	0.872039	{0.47755, 1.46064}	38.6379	ar	863.	921.5	{900., 940.}	6.77868
37	R0	{2.29641, 16.9868}	{2.26229, 19.3811}	{{2.26161, 19.3849}, {2.26161, 19.3849}}	{1.48599, 14.0949}	inf	186.	147.821	{89., 194.}	22.5038	0.127169	newly	65.	49.8571	{25., 72.}	27.0879	0.159462	Reff	0.803743	0.770626	{0.438752, 1.29138}	35.4027	ar	877.	860.232	{836., 884.}	2.24181
38	R0	{2.65743, 15.4569}	{2.82476, 19.9}	{{2.81635, 19.898}, {2.85956, 19.8999}}	{6.29675, 28.7453}	inf	233.	204.18	{159., 275.}	18.9263	0.0995947	newly	114.	66.459	{45., 91.}	41.7026	0.25148	Reff	0.733451	0.68875	{0.367473, 1.01107}	41.9578	ar	897.	925.689	{909., 942.}	3.22386
39	R0	{2.67953, 14.5132}	{2.9699, 18.3609}	{{2.90165, 18.0404}, {2.99088, 18.9406}}	{10.8367, 26.5116}	inf	233.	240.264	{187., 294.}	15.8858	0.0683816	newly	117.	84.2222	{55., 112.}	28.3476	0.157067	Reff	1.15488	0.697829	{0.36506, 0.995963}	46.9746	ar	894.	935.556	{920., 948.}	4.67934
40	R0	{1.94594, 11.928}	{2.81415, 15.8804}	{{2.688, 14.8279}, {2.93371, 17.1903}}	{44.6163, 33.1355}	inf	115.	143.859	{85., 235.}	40.3552	0.145315	newly	66.	56.6197	{32., 95.}	31.4554	0.160723	Reff	0.8387	0.41802	{0.26399, 0.637804}	53.3082	ar	796.	924.394	{908., 940.}	16.1299
41	R0	{1.66297, 10.0047}	{2.2679, 19.2873}	{{2.26161, 19.0355}, {2.29738, 19.3849}}	{36.3763, 92.7825}	inf	73.	159.5	{94., 210.}	121.575	0.335714	newly	55.	55.5357	{32., 76.}	26.4935	0.128241	Reff	0.964523	0.815917	{0.481723, 1.27505}	32.6751	ar	716.	857.411	{832., 884.}	19.7501
42	R0	{2.91568, 16.4453}	{2.85467, 14.3848}	{{2.81617, 13.3345}, {2.86193, 14.6465}}	{2.32464, 12.5295}	inf	275.	207.524	{134., 271.}	25.0563	0.139669	newly	105.	90.8254	{59., 124.}	23.5979	0.121557	Reff	0.906776	0.641169	{0.366327, 0.950161}	45.4272	ar	919.	928.206	{908., 944.}	1.70993
43	R0	{2.55898, 15.8889}	{2.86026, 16.7251}	{{2.78517, 15.7559}, {2.98558, 18.4042}}	{11.7735, 6.54126}	inf	219.	249.176	{184., 300.}	22.0597	0.0927904	newly	75.	99.8108	{75., 118.}	36.3604	0.133844	Reff	1.07989	1.01731	{0.537538, 1.66275}	37.5425	ar	904.	930.392	{912., 946.}	2.93441
44	R0	{2.28649, 15.9017}	{2.9332, 18.6922}	{{2.86501, 18.1089}, {2.99088, 18.9406}}	{28.284, 17.5482}	inf	219.	237.946	{182., 287.}	17.0924	0.0713613	newly	76.	83.8243	{55., 108.}	22.4929	0.0916194	Reff	0.756828	0.772205	{0.410077, 1.34637}	39.0783	ar	880.	933.676	{916., 948.}	6.09951
45	R0	{2.65121, 18.6339}	{2.88207, 14.786}	{{2.81617, 13.9425}, {2.96481, 15.2798}}	{8.80229, 20.6502}	inf	260.	247.485	{191., 300.}	12.845	0.0627418	newly	105.	108.956	{80., 138.}	15.4482	0.0671067	Reff	1.01011	0.841381	{0.471405, 1.41379}	38.8655	ar	928.	932.515	{919., 948.}	1.12037
46	R0	{2.52291, 18.0251}	{2.85051, 19.8443}	{{2.81635, 19.898}, {2.93978, 19.9817}}	{12.9851, 10.2124}	inf	228.	206.053	{131., 270.}	20.1177	0.100696	newly	79.	69.1579	{43., 94.}	22.8514	0.11709	Reff	0.915816	0.665542	{0.352044, 1.17297}	48.5262	ar	894.	926.987	{913., 942.}	3.6898
47	R0	{2.22618, 17.1225}	{2.84136, 19.0168}	{{2.65407, 17.2462}, {2.93978, 19.9817}}	{27.634, 12.0464}	inf	176.	260.634	{200., 309.}	51.8646	0.181886	newly	67.	92.831	{69., 110.}	41.0763	0.14676	Reff	1.15316	1.03737	{0.558979, 1.54221}	32.8362	ar	850.	929.254	{909., 945.}	9.32394
48	R0	{2.04405, 16.073}	{2.63456, 16.2501}	{{2.42221, 13.5694}, {2.88523, 18.4275}}	{28.8891, 10.4205}	inf	137.	191.681	{129., 250.}	44.9615	0.15942	newly	68.	77.7083	{52., 106.}	27.308	0.110444	Reff	1.11196	0.742158	{0.419659, 1.25785}	44.6852	ar	779.	901.958	{869., 931.}	15.7841
49	R0	{2.84396, 18.097}	{2.90067, 12.8448}	{{2.79531, 12.1249}, {2.95209, 13.7076}}	{2.67883, 29.0226}	inf	289.	159.648	{99., 237.}	45.1484	0.2839	newly	115.	73.3521	{43., 111.}	40.2082	0.238425	Reff	0.665487	0.422774	{0.281943, 0.585849}	41.8161	ar	952.	930.07	{908., 947.}	2.3272
50	R0	{1.85341, 18.0975}	{2.22146, 10.2185}	{{2.03307, 8.59564}, {2.38758, 11.4067}}	{19.8582, 43.5362}	inf	148.	30.3651	{8., 64.}	79.4831	0.849676	newly	54.	16.9206	{5., 38.}	69.8413	0.664293	Reff	0.850715	0.377313	{0.2981, 0.477771}	55.6475	ar	754.	847.095	{791., 888.}	12.3468
